# Supplementary material for: Peptide-Based Vaccine against SARS-CoV-2: Peptide Antigen Discovery and Screening of Adjuvant Systems
Source: Pharmaceutics. 2022 Apr 13;14(4):856. doi: 10.3390/pharmaceutics14040856 (PMC9024957; doi:10.3390/pharmaceutics14040856)
Supplement: Supplementary file 1 [file pharmaceutics-14-00856-s001.zip › pharmaceutics-1637636-supplementary.pdf]

pharmaceutics

MDPI

Supplementary Materials:

Peptide-based Vaccine against SARS-CoV-2: Peptide Antigen Discovery and Screening of Adjuvant Systems

Ahmed O. Shalash, Armira Azuar, Harrison Y. R. Madge, Naphak Modhiran, Alberto A. Amarilla, Benjamin Liang, Alexander A. Khromykh, Waleed M. Hussein, Keith J. Chappell, Daniel Watterson, Paul R. Young, Mariusz Skwarczynski, and Istvan Toth

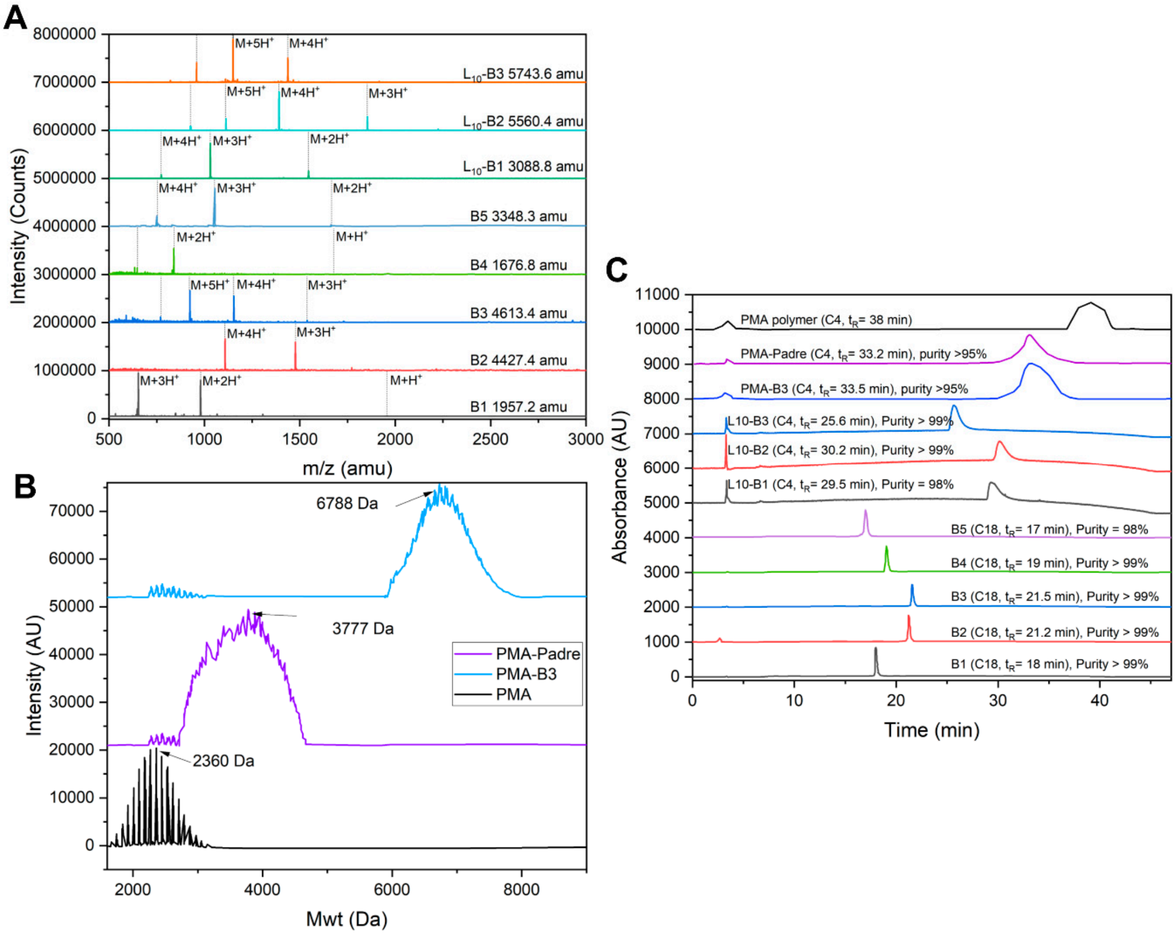

Figure S1. Characterization results of synthesized peptides and peptide conjugates by MS-ESI (A), MALDI-ToF (B), and RP-HPLC (C).

320 330 340 350
RV QPTESIVRFP NITNLCPFGE VFNATRFASV

360 370 380 390 400
YAWNRKRISN CVADY**SVLYN SASFSTFKCY GVSPTKLNDL CFTNVY**ADSF
------------**B5** (S^{366}-S^{395})-------------
410 420 430 440 450
VIRGDEVRQI APGQTGKIAD YNYKLPDDFT GCVIAWNSNN LDSK**VGGNYN**
----
460 470 480 490 500
YLYRLFRKSN LKPFERDI*ST EIYQAGSTPC NGV*EGFNCYF PLQSYGFQPT
-----**B3** (S^{444}-S^{483})-----------------------**B2** (S^{469}-S^{508})-----
510 520 530 540 550
NGVGYQPYRV VVLSFELLHA PATVCGPKKS TNLVKNKCVN FNFNGLTGTG
----
560 570 580 590 600
VLTESNKK**FL PFQQFGRDIA DT**TDAVRDPQ TLEILDITPC SFGGVSVITP
--**B4** (S^{559}-S^{572})---
610 620 630 640
GTNTSNQVAV LYQDVNCTEV PV**AIHADQLT PTWRVYSTG**S
--- **B1** (S^{623}-S^{639})---

Figure S2. Peptide antigen sequences B1-B5 (bolded) and their locations on the original Wuhan-1 strain SARS-CoV-2 spike protein sequence with RBD amino acid sequence coloured in blue. The italicized sequence "*STEIYQAGSTPCNGV*" is a part of both B2 and B3 sequences.

Table S1. Secondary structural content of screened and candidate peptide B-cell epitopes.

Peptide	***Experimental***			***i-tasser predicted*** [a]			*Native (Cryo-EM)* [a]		
	**Helix**	**Strand**	**Random Coil**	**Helix**	**Strand**	Random Coil	Helix	Strand	Random Coil
	%	%	%	%	%	%	%	%	%
B1	28.5	2.5	69.0	47.0	0.0	53.0	--	--	--
B2	18.0	20.5	61.5	7.5	22.5	70.0	0.0	10.0	90.0
B3	29.0	0.0	71.0	59.0	0.0	41.0	0.0	10.0	90.0
B4	27.0	0.0	73.0	43.0	0.0	57.0	0.0	17.5	82.5
B5	0.0	59.5	40.5	10.0	20.0	70.0	13.0	13.0	74.0
L_{10}-B1	18.0	82.0	0.0	35.0	23.5	41.5		Non-native	
L_{10}-B2	95.0	0.0	5.0	22.5	0.0	77.5		Non-native	
L_{10}-B3	61.0	23.0	16.0	59.0	0.0	41.0		Non-native	

[a] Predicted percentage results were calculated by summing the amino acid residues of a specific secondary structure, then dividing that by the total number of residues in the peptide [1,70].

The L10-conjugated peptide antigens were self-assembled in PBS, resulting in microparticles (Figure S2). L10-B1, L10-B2, and L10-B3 self-assmebled into particles with sizes of: 1.42 ± 0.12 μm, 1.32 ± 0.08 μm, and 1.38 ± 0.17 μm, respectively, with relatively small polydispersity indices (PDIs) for aggregating, self-assmebled particles (PDI = 0.302 ± 0.072, 0.265 ± 0.078, and 0.398 ± 0.022, respectively).

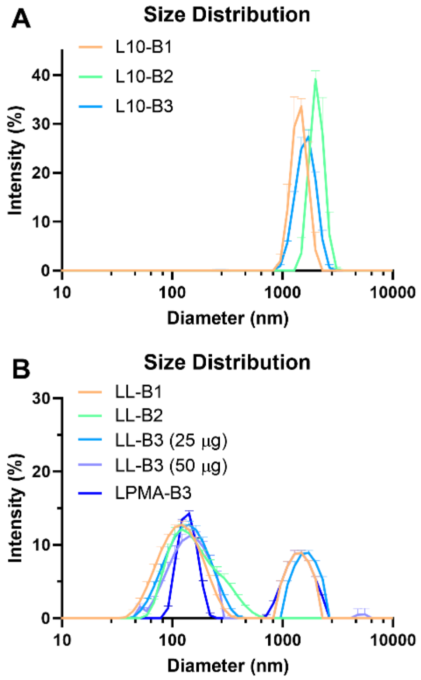

Figure S3. Size distribution of (**A**) self-assembled peptide antigens, and (**B**) liposomal formulations of polyleucine-, or PMA- conjugated peptides.

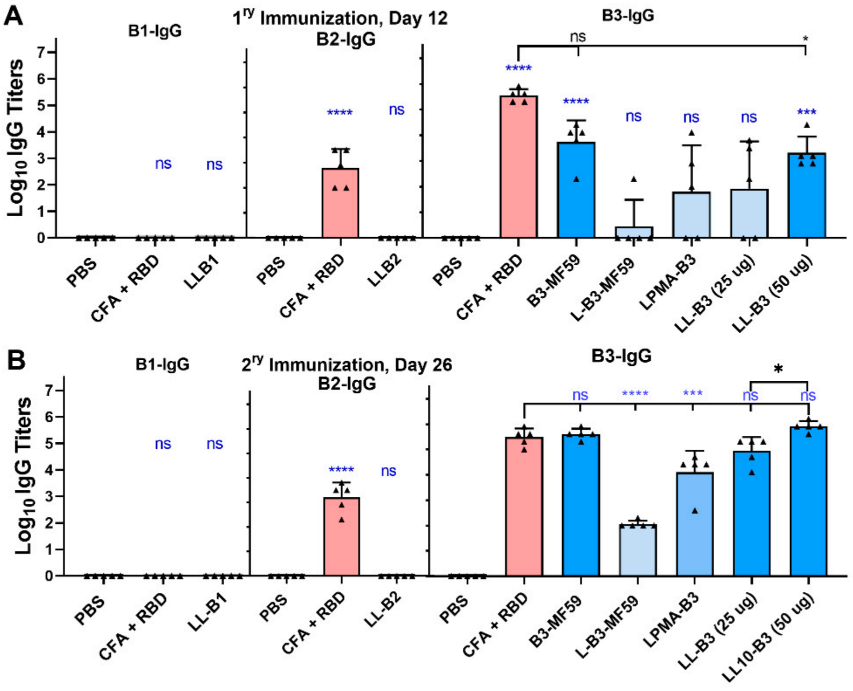

Figure S4. Immunogenicity results from the peptide adjuvant screening study following immunization of C57BL/6 mice. Anti-peptide IgG titers in the serum after primary immunization (**A**), and anti-peptide IgG titers against self-peptide antigens B1–B3 after secondary immunization (**B**). Error bars represent standard deviation. Statistical analysis was performed using one-way ANOVA with Dunnett's multiple comparison test. (ns) non-significant, $p > 0.05$; (*) $p < 0.05$; (**) $p < 0.01$; (***) $p < 0.001$, and (****) $p < 0.0001$.

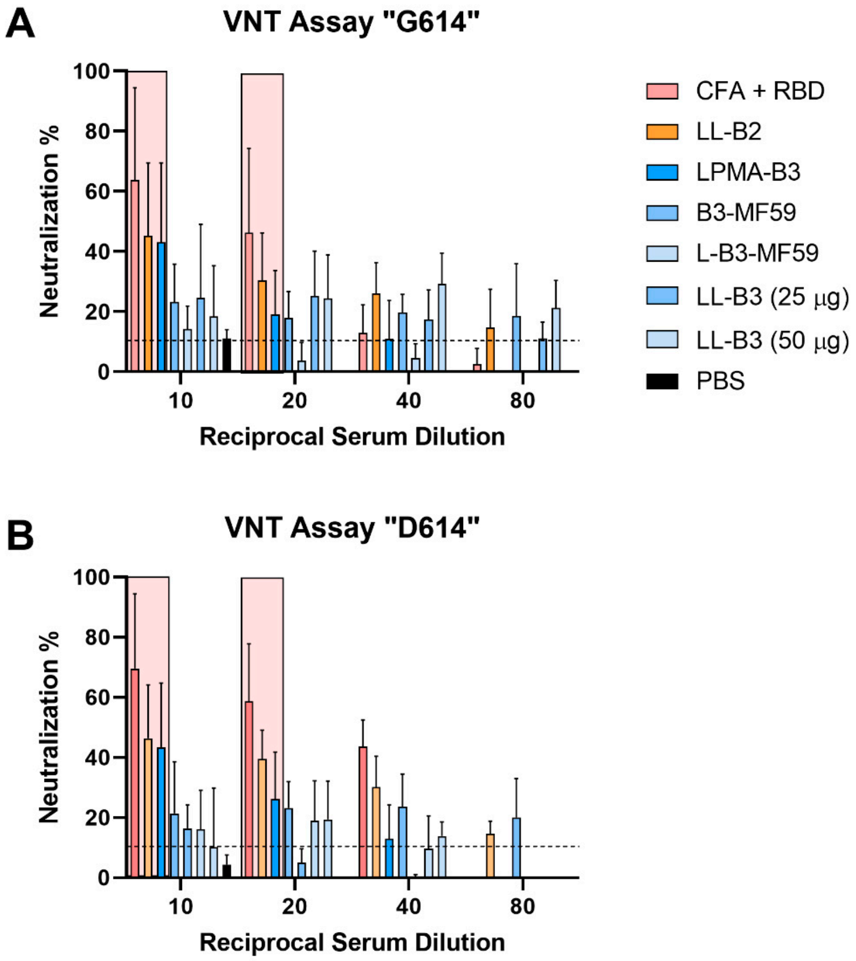

Figure S5. Live virus neutralization assays of mouse serum against the original SARS-CoV-2 Wuhan strain (G614) (**A**), and the D614 variant (**B**). Red boxes highlight the most promising neutralizing sera.
